# Supplementary material for: Socio-demographic and genetic risk factors for drug adherence and persistence across 5 common medication classes
Source: Nat Commun. 2024 Oct 23;15:9156. doi: 10.1038/s41467-024-53556-z (PMC11500092; doi:10.1038/s41467-024-53556-z)
Supplement: Supplementary file 2 — Description of Additional Supplementary Files [file 41467_2024_53556_MOESM2_ESM.pdf]

Description of Additional Supplementary Files

File Name: Supplementary Data 1

Description: Inclusion criteria for adherence and persistence analysis and number of individuals included for each medication in the Finnish population. For each medication, we report the total number of individuals with at least one purchase, and the number of individuals remaining after applying each of the inclusion criteria, reported in each column. The last row reports the total number of unique individuals across all medications classes considered.

File Name: Supplementary Data 2

Description: Descriptive statistics of persistence and adherence for the 8 health, demographic and socio-economic factors considered in the study. Per each class of medication and within each factor level, the table reports the total number of individuals considered in the study, the proportion of persistent individuals, the mean adherence (and standard deviation).

File Name: Supplementary Data 3

Description: Associations between persistence and 8 health, demographic and socioeconomic factors. Results from a multivariate logistic regression model including the 8 factors (keeping continuous variables as such) and adjusted for year of birth. P values are two-sided and were calculated by dividing the coefficient values by their standard errors and observing the probability mass corresponding to equal or more extreme values from both tails of the standard normal distribution. No adjustments for multiple comparisons were made.

File Name: Supplementary Data 4

Description: Associations between adherence and 8 health, demographic and socioeconomic factors. Results from a multivariate linear regression model including the 8 factors (keeping continuous variables as such) and adjusted for year of birth. Percentage change in adherence is the beta estimate from the model, rescaled for the maximum adherence value of 1.1. P values are two-sided and were calculated by dividing the coefficient values by their standard errors and observing the probability mass corresponding to equal or more extreme values from both tails of the t-distribution. No adjustments for multiple comparisons were made.

File Name: Supplementary Data 5

Description: Associations between persistence and 8 health, demographic and socioeconomic factors (continuous variables binarized). Results from a multivariate logistic regression model including the 8 factors and adjusted for year of birth. Continuous variables were binarized at the specified levels. P values are two-sided and were calculated by dividing the coefficient values by their standard errors and observing the probability mass corresponding to equal or more extreme values from both tails of the standard normal distribution. No adjustments for multiple comparisons were made

File Name: Supplementary Data 6

Description: Associations between adherence and 8 health, demographic and socioeconomic factors (continuous variables binarized). Results from a multivariate linear regression model including the 8 factors and adjusted for year of birth. Continuous variables were binarized at the specified levels. Percentage change in adherence is the beta estimate from the model, rescaled for the maximum adherence value of 1.1. P values are two-sided and were calculated by dividing the coefficient values by their standard errors and observing the probability mass corresponding to equal or more extreme values from both tails of the t-distribution. No adjustments for multiple comparisons were made.

File Name: Supplementary Data 7

Description: Associations between persistence and polytherapy. Results from a multivariate logistic regression model including the number of concurrent treatments and adjusting for the factors used in our primary analysis. The concurrent treatment variable was categorized with three levels (0 for no concurrent treatment, 1 for one concurrent treatment, and 2 for more than one concurrent treatment). P values are two-sided and were calculated by dividing the coefficient values by their standard errors and observing the probability mass corresponding to equal or more extreme values from both tails of the standard normal distribution. No adjustments for multiple comparisons were made

File Name: Supplementary Data 8

Description: Associations between adherence and polytherapy. Results from a multivariate linear regression model including the number of concurrent treatments and adjusting for the factors used in our primary analysis. The concurrent treatment variable was categorized with three levels (0 for no concurrent treatment, 1 for one concurrent treatment, and 2 for more than one concurrent treatment). Percentage change in adherence is the beta estimate from the model, rescaled for the maximum adherence value of 1.1. P values are two-sided and were calculated by dividing the coefficient values by their standard errors and observing the probability mass corresponding to equal or more extreme values from both tails of the t-distribution. No adjustments for multiple comparisons were made.

File Name: Supplementary Data 9

Description: Comparison of variance explained by the multivariable model for persistence and adherence. Adjusted McFadden pseudo R2 were calculated comparing the log

likelihood of the full multivariable logistic model including the 8 risk factors and the log likelihood of a model including only the intercept. Adherence phenotype has been binarised in good (>0.8) vs bad adherence to allow the comparison with persistence. P values are two-sided and were calculated by dividing the coefficient values by their standard errors and observing the probability mass corresponding to equal or more extreme values from both tails of the t-distribution. No adjustments for multiple comparisons were made.

File Name: Supplementary Data 10

Description: Association between persistence and adherence to statins and demographic factors in Estonian Biobank. Results from, respectively, a logistic and linear regression models including the predictors common to FinRegistry and Estonian Biobank. P values are two-sided and were calculated by dividing the coefficient values by their standard errors and observing the probability mass corresponding to equal or more extreme values from both tails of the relevant distribution (standard normal distribution for logistic regression and t-distribution for linear regression). No adjustments for multiple comparisons were made.

File Name: Supplementary Data 11

Description: Association between persistence and relevant pharmacogenes. Association between persistence in taking statins and tamoxifen and drug-specific relevant metabolic phenotypes defined based on pharmacogenes diplotypes. Coefficients are from a logistic regression model, adjusting for sex, age at initiation and first 10 genetic principal components. Phenotype classes with count < 5 have not been tested. P values are two-sided and were calculated by dividing the coefficient values by their standard errors and observing the probability mass corresponding to equal or more extreme values from both tails of the standard normal distribution. No adjustments for multiple comparisons were made.

File Name: Supplementary Data 12

Description: Association between adherence and relevant pharmacogenes. Association between adherence to statins, clopidogrel and tamoxifen and drug-specific relevant metabolic phenotypes defined based on pharmacogenes diplotypes. Coefficients are from a linear regression model, adjusting for sex, age at initiation and first 10 genetic principal components. Phenotype classes with count < 5 have not been tested. P values are two-sided and were calculated by dividing the coefficient values by their standard errors and observing the probability mass corresponding to equal or more extreme values from both tails of the t-distribution. No adjustments for multiple comparisons were made.

File Name: Supplementary Data 13

Description: Genome wide association results for persistence and adherence. Lead variants for the loci that showed a genome-wide significant (P < 5 × 10-8) association with persistence or adherence to the medications considered.

File Name: Supplementary Data 14

Description: External GWAS resources used in genetic correlation and PGS analyses.

File Name: Supplementary Data 15

Description: Genetic correlates of adherence and persistence in the Finnish population. Genetic correlations between 33 clinically relevant traits and persistence and adherence, derived using linkage disequilibirum score regression. Medications for which genetic correlations could not be computed are not reported. Two-sided P values were calculated using LD Score Regression.

File Name: Supplementary Data 16

Description: Associations between persistence and PGS for 33 clinically relevant traits in the Finnish population. Results from a logistic regression model including the standardized PGS and adjusted for sex, age at initiation, first 10 genetic principal components. Last column reports the coefficient estimate as odds of persistence per 1-SD increase in trait PGS. P values are two-sided and were calculated by dividing the coefficient values by their standard errors and observing the probability mass corresponding to equal or more extreme values from both tails of the standard normal distribution. No adjustments for multiple comparisons were made.

File Name: Supplementary Data 17

Description: Associations between adherence and PGS for 33 clinically relevant traits in the Finnish population. Results from a linear regression model including the standardized PGS and adjusted for sex, age at initiation, first 10 genetic principal components. Last column reports the coefficient estimate as percentage change in adherence per 1-SD increase in trait PGS. P values are two-sided and were calculated by dividing the coefficient values by their standard errors and observing the probability mass corresponding to equal or more extreme values from both tails of the t-distribution. No adjustments for multiple comparisons were made.

File Name: Supplementary Data 18

Description: Associations between persistence and PGS for 33 clinically relevant traits in the Estonian Biobank. Results from a logistic regression model including the standardized PGS and adjusted for sex, age at initiation, first 10 genetic principal components. Last column reports the coefficient estimate as odds of persistence per 1-SD increase in trait PGS. P values are two-sided and were calculated by dividing the coefficient values by their standard errors and observing the probability mass corresponding to equal or more extreme values from both tails of the standard normal distribution. No adjustments for multiple comparisons were made.

File Name: Supplementary Data 19

Description: Associations between adherence and PGS for 33 clinically relevant traits in the Estonian Biobank. Results from a logistic regression model including the standardized PGS and adjusted for sex, age at initiation, first 10 genetic principal components. Last column reports the coefficient estimate as odds of persistence per 1-SD increase in trait PGS. P values are two-sided and were calculated by dividing the coefficient values by their standard errors and observing the probability mass corresponding to equal or more extreme values from both tails of the t-distribution. No adjustments for multiple comparisons were made.

File Name: Supplementary Data 20

Description: Comparison for variance in adherence explained by multivariable epidemiological model and selected PGS.

File Name: Supplementary Data 21

Description: Associations between persistence (as defined for the sensitivity analysis) and PGS for 33 clinically relevant traits. Results from a logistic regression model including the standardized PGS and adjusted for sex, age at initiation, first 10 genetic principal components. Last column reports the coefficient estimate as odds of persistence per 1-SD increase in trait PGS. P values are two-sided and were calculated by dividing the coefficient values by their standard errors and observing the probability mass corresponding to equal or more extreme values from both tails of the standard normal distribution. No adjustments for multiple comparisons were made.
