# Supplementary material for: Socio-demographic and genetic risk factors for drug adherence and persistence across 5 common medication classes
Source: Nat Commun. 2024 Oct 23;15:9156. doi: 10.1038/s41467-024-53556-z (PMC11500092; doi:10.1038/s41467-024-53556-z)
Supplement: Supplementary file 4 — Reporting Summary [file 41467_2024_53556_MOESM4_ESM.pdf]

nature portfolio

Corresponding author(s): Andrea Ganna

Last updated by author(s): Sep 21, 2024

Reporting Summary

Nature Portfolio wishes to improve the reproducibility of the work that we publish. This form provides structure for consistency and transparency in reporting. For further information on Nature Portfolio policies, see our Editorial Policies and the Editorial Policy Checklist.

Statistics

For all statistical analyses, confirm that the following items are present in the figure legend, table legend, main text, or Methods section.

n/a	Confirmed	
☐	☒	The exact sample size (n) for each experimental group/condition, given as a discrete number and unit of measurement
☐	☒	A statement on whether measurements were taken from distinct samples or whether the same sample was measured repeatedly
☐	☒	The statistical test(s) used AND whether they are one- or two-sided<br>*Only common tests should be described solely by name; describe more complex techniques in the Methods section.*
☐	☒	A description of all covariates tested
☐	☒	A description of any assumptions or corrections, such as tests of normality and adjustment for multiple comparisons
☐	☒	A full description of the statistical parameters including central tendency (e.g. means) or other basic estimates (e.g. regression coefficient) AND variation (e.g. standard deviation) or associated estimates of uncertainty (e.g. confidence intervals)
☐	☒	For null hypothesis testing, the test statistic (e.g. F, t, r) with confidence intervals, effect sizes, degrees of freedom and P value noted<br>*Give P values as exact values whenever suitable.*
☒	☐	For Bayesian analysis, information on the choice of priors and Markov chain Monte Carlo settings
☒	☐	For hierarchical and complex designs, identification of the appropriate level for tests and full reporting of outcomes
☐	☒	Estimates of effect sizes (e.g. Cohen's d, Pearson's r), indicating how they were calculated

Our web collection on statistics for biologists contains articles on many of the points above.

Software and code

Policy information about availability of computer code

Data collection: No software was used for data collection.

Data analysis: All analysis steps and related tools are described in Methods. The softwares used, and their versions, are:
R v4
ICCI R package https://github.com/dsgelab/ICCI
Stargazer v2 https://stargazer.gs.washington.edu/stargazerweb/index.html
REGENIE v2.2.4 https://doi.org/10.1038/s41588-021-00870-7
LDSC v.10.1 https://github.com/bulik/ldsc
PRS-CS https://github.com/getian107/PRScs

Analysis code used to produce the results is available on GitHub at: https://github.com/dsgelab/drugs-persistence-adherence

For manuscripts utilizing custom algorithms or software that are central to the research but not yet described in published literature, software must be made available to editors and reviewers. We strongly encourage code deposition in a community repository (e.g. GitHub). See the Nature Portfolio guidelines for submitting code & software for further information.

Data

Policy information about availability of data

All manuscripts must include a data availability statement. This statement should provide the following information, where applicable:

- Accession codes, unique identifiers, or web links for publicly available datasets
- A description of any restrictions on data availability
- For clinical datasets or third party data, please ensure that the statement adheres to our policy

Data dictionaries for FinRegistry are publicly available on the FinRegistry website (www.finregistry.fi/finnish-registry-data). Access to the FinRegistry data can be obtained by submitting a data permit application for individual-level data to the Finnish social and health data permit authority, Findata (https://asiointi.findata.fi/). The application includes information on the purpose of data use; the requested data, including the variables, definitions of the target and control groups, and external datasets to be combined with FinRegistry data; the dates of the data needed; and a data utilization plan. The requests are evaluated case by case. Once approved, the data are sent to a secure computing environment (Kapseli) and can be accessed within the European Economic Area and within countries with an adequacy decision from the European Commission. The Finnish biobank data can be accessed through the Fingenious services (https://site.fingenious.fi/en/) managed by FINBB. Access to individual level data from the Estonian Biobank can be obtained through the Estonian Biobank, following standard data access procedures (https://genomics.ut.ee/en/content/estonian-biobank). All research using the Estonian Biobank data is regulated by the Human Genes Research Act and must be approved by the Estonian Committee of Bioethics and Human Research. Summary statistics for the GWAS of adherence and persistence to statins and blood pressure medications are available in the GWAS catalogue under accession codes GCST90448318, GCST90448319, GCST90448320, GCST90448321.

Research involving human participants, their data, or biological material

Policy information about studies with human participants or human data. See also policy information about sex, gender (identity/presentation), and sexual orientation and race, ethnicity and racism.

Reporting on sex and gender	Sex was used as covariate in all of the analyses and no analyses were run separately in each sex. In FinRegistry, sex was defined as recorded in the population registries from the Digital and Population Data Services Agency. In FinnGen, genetically determined sex was used, after quality control removal for individuals with discrepancy between sex reported in the population registries and genetically determined sex. In the Estonian Biobank, sex was extracted from the Estonian National Identity number which is created based on the sex recorded in the Estonian Birth Registry. Quality assurance was carried out where only individuals whose sex from the National Identity number matched the sex in genotype data are included.
Reporting on race, ethnicity, or other socially relevant groupings	Mother tongue was categorised as Finnish/Swedish (the two official languages of Finland) vs other. This variable was used as deemed relevant in studying adherence to recommendation from health care providers.<br><br>Receiving any kind of social assistance benefit in the year before starting a drug treatment was used as a proxy for lower socio-economic status, and deemed relevant as it might impact access to healthcare.
Population characteristics	The FinRegistry dataset contains data from Finnish nationwide health, demographic, and socioeconomic registries, for a total of 7 166 416 individuals. To ensure completeness of information for the variables considered in the epidemiological analysis, we included only individuals who were residents in Finland and alive on 1 January 2010 (5 339 804 individuals in total). The FinnGen dataset additionally contains genetic data for a subset of the Finnish population (N = 430 885 for data freeze 10). The Estonian Biobank is a volunteer-based biobank that has a sample size of approximately 207,000 participants, comprising over 20% of the adult population of Estonia. The EstBB is linked with the National Health Insurance Fund's (NHIF) database which was used as the EHR source. We studied the two drug usage phenotypes in adults only, meaning individuals needed to be at least 18 years old at treatment initiation.
Recruitment	The Finregistry dataset includes each individual alive and living in Finland on 1.1.2010.
Ethics oversight	FinRegistry is a collaboration project of the Finnish Institute for Health and Welfare (THL) and the Data Science Genetic Epidemiology research group at the Institute for Molecular Medicine Finland (FIMM), University of Helsinki. The FinRegistry project has received the following approvals for data access from the National Institute of Health and Welfare (THL/1776/6.02.00/2019 and subsequent amendments), DVV (VRK/5722/2019-2), Finnish Center for Pension (ETK/SUTI 22003) and Statistics Finland (TK-53-1451-19). The FinRegistry project has received IRB approval from the National Institute of Health and Welfare (Kokous 7/2019).<br>Patients and control subjects in FinnGen provided informed consent for biobank research, based on the Finnish Biobank Act. Alternatively, separate research cohorts, collected prior the Finnish Biobank Act came into effect (in September 2013) start of FinnGen (August 2017), were collected based on study-specific consents and later transferred to the Finnish biobanks after approval by Fimea (Finnish Medicines Agency), the National Supervisory Authority for Welfare and Health. Recruitment protocols followed the biobank protocols approved by Fimea. The Coordinating Ethics Committee of the Hospital District of Helsinki and Uusimaa (HUS) statement number for the FinnGen study is Nr HUS/990/2017.<br>The FinnGen study is approved by Finnish Institute for Health and Welfare (permit numbers: THL/2031/6.02.00/2017, THL/1101/5.05.00/2017, THL/341/6.02.00/2018, THL/2222/6.02.00/2018, THL/283/6.02.00/2019, THL/1721/5.05.00/2019 and THL/1524/5.05.00/2020), Digital and population data service agency (permit numbers: VRK/6909/2018-3, VRK/4415/2019-3), the Social Insurance Institution (permit numbers: 58/522/2017, KELA 131/522/2018, KELA 70/522/2019, KELA 98/522/2019, KELA 134/522/2019, KELA 138/522/2019, KELA 2/522/2020, KELA 16/522/2020), Findata permit numbers THL/2364/14.02/2020, THL/4055/14.06.00/2020, THL/3433/14.06.00/2020, THL/4432/14.06/2020, THL/5189/14.06/2020, THL/5894/14.06.00/2020, THL/6619/14.06.00/2020, THL/209/14.06.00/2021, THL/688/14.06.00/2021, THL/1284/14.06.00/2021, THL/1965/14.06.00/2021, THL/5546/14.02.00/2020, THL/2658/14.06.00/2021, THL/4235/14.06.00/2021, Statistics Finland (permit numbers: TK-53-1041-17 and TK/143/07.03.00/2020 (earlier TK-53-90-20)

VRK43431/2017-3,
KELA
TK/1735/07.03.00/2021, TK/3112/07.03.00/2021) and Finnish Registry for Kidney Diseases permission/extract from the meeting minutes on 4th July 2019.
The Biobank Access Decisions for FinnGen samples and data utilized in FinnGen Data Freeze 10 include: THL Biobank BB2017_55, BB2017_111, BB2018_19, BB_2018_34, BB_2018_67, BB2018_71, BB2019_7, BB2019_8, BB2019_26, BB2020_1, BB2021_65, Finnish Red Cross Blood Service Biobank 7.12.2017, Helsinki Biobank HUS/359/2017, HUS/248/2020, HUS/150/2022 § 12, §13, §14, §15, §16, §17, §18, and §23, Auria Biobank AB17-5154 and amendment #1 (August 17 2020) and amendments BB_2021-0140, BB_2021-0156 (August 26 2021, Feb 2 2022), BB_2021-0169, BB_2021-0179, BB_2021-0161, AB20-5926 and amendment #1 (April 23 2020)and it ́s modification (Sep 22 2021), Biobank Borealis of Northern Finland_2017_1013, 2021_5010, 2021_5018, 2021_5015, 2021_5023, 2021_5017, 2022_6001, Biobank of Eastern Finland 1186/2018 and amendment 22 § /2020, 53§/2021, 13§/2022, 14§/2022, 15§/2022, Finnish Clinical Biobank Tampere MH0004 and amendments (21.02.2020 & 06.10.2020), §8/2021, §9/2022, §10/2022, §12/2022, §20/2022, §21/2022, §22/2022, §23/2022, Central Finland Biobank 1-2017, and Terveystalo Biobank STB 2018001 and amendment 25th Aug 2020, Finnish Hematological Registry and Clinical Biobank decision 18th June 2021, Arctic biobank P0844: ARC_2021_1001.
The activities of the Estonian Biobank (EstBB) are regulated by the Human Genes Research Act, which was adopted in 2000 specifically for the operations of the EstBB. The analysis of individual-level data from the Estonian Biobank was carried out under ethical approval nr 1.1-12/624 from the Estonian Committee on Bioethics and Human Research (Estonian Ministry of Social Affairs), using data according to data release application nr T12 6-7/GI/11086 from the Estonian Biobank.

Note that full information on the approval of the study protocol must also be provided in the manuscript.

Field-specific reporting

Please select the one below that is the best fit for your research. If you are not sure, read the appropriate sections before making your selection.

☒ Life sciences ☐ Behavioural & social sciences ☐ Ecological, evolutionary & environmental sciences

For a reference copy of the document with all sections, see nature.com/documents/nr-reporting-summary-flat.pdf

Life sciences study design

All studies must disclose on these points even when the disclosure is negative.

Sample size	The FinRegistry dataset contains data from Finnish nationwide health, demographic, and socioeconomic registries, for a total of 7 166 416 individuals. To ensure completeness of information for the variables considered in the epidemiological analysis, we included only individuals who were residents in Finland and alive on 1 January 2010 (5 339 804 individuals in total). The FinnGen dataset additionally contains genetic data for a subset of the Finnish population (N = 430 885 for data freeze 10). We studied the two drug usage phenotypes in adults only, meaning individuals needed to be at least 18 years old at treatment initiation.
Data exclusions	The following criteria were used for exclusion:<br>- individuals not purchasing the medications considered<br>- individuals younger than 18 at treatment initiation<br>- indiviudals not respecting specific phenotype definition criteria, as reported in Methods and Supplementary Table 1
Replication	We replicated all our analyses in Estonian Biobank for statin adherence and persistence. Additionally, we replicated the pharmacogenetic analyses for statins, clopidogrel and tamoxifen.
Randomization	Not relevant, as no division into cases and controls was considered.
Blinding	Not relevant, as the study does not consider allocation into case and control groups

Reporting for specific materials, systems and methods

We require information from authors about some types of materials, experimental systems and methods used in many studies. Here, indicate whether each material, system or method listed is relevant to your study. If you are not sure if a list item applies to your research, read the appropriate section before selecting a response.

Materials & experimental systems

n/a	Involved in the study	
☒	☐	Antibodies
☒	☐	Eukaryotic cell lines
☒	☐	Palaeontology and archaeology
☒	☐	Animals and other organisms
☒	☐	Clinical data
☒	☐	Dual use research of concern
☒	☐	Plants

Methods

n/a	Involved in the study	
☒	☐	ChIP-seq
☒	☐	Flow cytometry
☒	☐	MRI-based neuroimaging

Plants

Seed stocks	*Report on the source of all seed stocks or other plant material used. If applicable, state the seed stock centre and catalogue number. If plant specimens were collected from the field, describe the collection location, date and sampling procedures.*
Novel plant genotypes	*Describe the methods by which all novel plant genotypes were produced. This includes those generated by transgenic approaches, gene editing, chemical/radiation-based mutagenesis and hybridization. For transgenic lines, describe the transformation method, the number of independent lines analyzed and the generation upon which experiments were performed. For gene-edited lines, describe the editor used, the endogenous sequence targeted for editing, the targeting guide RNA sequence (if applicable) and how the editor was applied.*
Authentication	*Describe any authentication procedures for each seed stock used or novel genotype generated. Describe any experiments used to assess the effect of a mutation and, where applicable, how potential secondary effects (e.g. second site T-DNA insertions, mosiacism, off-target gene editing) were examined.*
